# Computational analysis for identification of early diagnostic biomarkers and prognostic biomarkers of liver cancer based on GEO and TCGA databases and studies on pathways and biological functions affecting the survival time of liver cancer

**DOI:** 10.1186/s12885-021-08520-1

**Published:** 2021-07-08

**Authors:** Shiyong Gao, Jian Gang, Miao Yu, Guosong Xin, Huixin Tan

**Affiliations:** 1 Drug Engineering and Technology Research Center, Harbin University of Commerce, Harbin, 150076 Heilongjiang China; 2 Heilongjiang Provincial Key Laboratory of Tumor Prevention and Antitumor Drugs, Harbin, 150076 Heilongjiang China; 3 Department of pharmacy, The Fourth Affiliated Hospital of Harbin Medicine University, Harbin, 150001 Heilongjiang China

**Keywords:** Bioinformatics, Biomarker, Liver cancer, Diagnostic biomarker, Prognostic biomarker

## Abstract

**Background:**

Liver cancer is the sixth most commonly diagnosed cancer and the fourth most common cause of cancer death. The purpose of this work is to find new diagnostic biomarkers or prognostic biomarkers and explore the biological functions related to the prognosis of liver cancer.

**Methods:**

GSE25097 datasets were firstly obtained and compared with TCGA LICA datasets and an analysis of the overlapping differentially expressed genes (DEGs) was conducted. Cytoscape was used to screen out the Hub Genes among the DEGs. ROC curve analysis was used to screen the Hub Genes to determine the genes that could be used as diagnostic biomarkers. Kaplan-Meier analysis and Cox proportional hazards model screened genes associated with prognosis biomarkers, and further Gene Set Enrichment Analysis was performed on the prognosis genes to explore the mechanism affecting the survival and prognosis of liver cancer patients.

**Results:**

790 DEGs and 2162 DEGs were obtained respectively from the GSE25097 and TCGA LIHC data sets, and 102 Common DEGs were identified by overlapping the two DEGs. Further screening identified 22 Hub Genes from 102 Common DEGs. ROC and survival curves were used to analyze these 22 Hub Genes and it was found that there were 16 genes with a value of AUC > 90%. Among these, the expression levels of ESR1,SPP1 and FOSB genes were closely related to the survival time of liver cancer patients. Three common pathways of ESR1, FOBS and SPP1 genes were identified along with seven common pathways of ESR1 and SPP1 genes and four common pathways of ESR1 and FOSB genes.

**Conclusions:**

SPP1, AURKA, NUSAP1, TOP2A, UBE2C, AFP, GMNN, PTTG1, RRM2, SPARCL1, CXCL12, FOS, DCN, SOCS3, FOSB and PCK1 can be used as diagnostic biomarkers for liver cancer, among which FOBS and SPP1 genes can also be used as prognostic biomarkers. Activation of the cell cycle-related pathway, pancreas beta cells pathway, and the estrogen signaling pathway, while on the other hand inhibition of the hallmark heme metabolism pathway, hallmark coagulation pathway, and the fat metabolism pathway may promote prognosis in liver cancer patients.

**Supplementary Information:**

The online version contains supplementary material available at 10.1186/s12885-021-08520-1.

## Background

According to the Global Cancer Statistics report of 2018, liver Cancer became the sixth most commonly diagnosed cancer and the fourth leading cause of cancer death in the world in 2018 [1]. The highest incidence (mortality) of liver cancer is in East Asia, accounting for 35.5% of the global total. The main risk factors for liver cancer are chronic hepatitis B virus (HBV) [2–4], hepatitis C virus (HCV) [5–7], aflatoxin-contaminated food [8], heavy alcohol consumption [6, 9, 10], obesity [11], smoking [12] and type 2 diabetes [13, 14]. According to statistics, the risk factors of liver cancer formation are different in 53 countries and throughout different regions in the world. In most high-risk areas such as China and East Africa, chronic HBV infection and aflatoxin exposure are the main determinants of liver cancer. In contrast, HCV infection is the leading cause of liver cancer in Japan and Egypt [15, 16]. For low-risk liver cancer areas, an increase in obesity rates is the leading cause of the increase in liver cancer case.

The internationally recognized TNM cancer staging method divides cancers into stage I, II, III and IV [17]. Also, work on the topic has previously divided cancer into early, middle and late stages. Corresponding to TNM stages, phase I is early-stage, phase II and III are middle-stages, and phase IV is late-stage. Most cancers are diagnosed at the late stage and this holds especially true for liver cancer. Modern medical research has shown that there is no pain sensation in the liver and even if any liver disease had started, the body can’t feel or recognize it through a pain-feedback mechanism. Hence, the clinical manifestation of liver disease is very slight, most patients with liver cancer are diagnosed at a late stage owing to a lack of timely symptom manifestation and identification [18–21]. The cure rate of early-stage liver cancer is very optimistic, therefore if a diagnosis can be made in any stage before stage IV, the treatment of the cancerous mass will be less intense as it would be for the final stage.

Alpha-fetoprotein (AFP) is currently the only clinically used biomarker for the early diagnosis of liver cancer. AFP was discovered more than 50 years ago and is not a very accurate diagnostic biomarker for liver cancer. 32 to 59% of liver cancer patients have been shown to have normal AFP levels [22]. Therefore, finding new diagnostic biomarkers of liver cancer is of great significance for accurate diagnosis. For cancer patients, the prognosis and survival time of cancer is of utmost importance for improving the quality of life of patients, as well as the diagnosis and treatment scheme adopted. Currently, therapeutic indications for the treatment of liver cancer are more concerned with tumor size and the number of nodules and less concerned with its aggressiveness to spread [23]. Compared with a small and aggressive liver cancer node, patients with multiple large but non aggressive liver cancer nodules may have a better prognosis, hence it may be assumed that the current prognostic criteria are not accurate or the best for prognosis. If new genes related to the prognosis of liver cancer can be identified, it will hold large positive ramifications for both treatment and the improvement of patients’ quality of life. In this scientific work, the data of liver cancer patients in TGCA and GEO databases were taken as search criteria to identify diagnostic biomarkers and prognostic biomarkers of liver cancer through data mining. The aim is to improve the accuracy of the early diagnosis of liver cancer, achieve early detection and treatment and thus reduce mortality. At the same time, through the accurate judgment of the prognosis of liver cancer patients, adjuvant treatment to determine the plan of action could be streamlined.

## Methods

### Microarray data

The liver cancer dataset was obtained from TCGA (https://portal.gdc.cancer.gov/). This included 50 normal liver tissue samples and 371 samples of liver cancer which was coupled with clinical data. Another gene expression profiling dataset (GSE25097) included information on 243 normal samples and 268 tumor samples, which was downloaded from Gene Expression Omnibus (GEO, https://www.ncbi.nlm.nih.gov/geo/database) and measured in an array (Platform: GPL10687 Rosetta/Merck Human RSTA Affymetrix 1.0 microarray, Custom CDF).

### Data processing

The original microarray data of the GSE25097 and TCGA LIHC datasets were respectively analyzed with R language to screen the differentially expressed genes (DEGs). Adj .*p*-value < 0.05 and |logFC| > 2 were used as the cut-off criteria. A Draw Venn Diagram online tool (http://bioinformatics.psb.ugent.be/webtools/Venn/) was used to calculate the intersection of two differentially DEGs derived from two different datasets, which represented common differentially expressed genes (the Common DEGs).

### Volcano maps and heat maps of DEGs obtained from GEO and TCGA databases

Packet pheatmap, packet ggplot2 and other R packets were used to draw heat maps and volcanic maps of DEGs.

### Gene ontology and Reactome pathway analysis

GO analysis of the obtained DEGs was carried out using the package clusterProfiler. The package ReactomePA was used for enrichment analysis of the obtained DEGs in the Reactome pathway. *P* < 0.05 was considered as statistically significant.

### Protein-protein interaction network

Search Tool for the Retrieval of Interacting Genes/Proteins (STRING) is an online protein interaction tool (https://string-db.org/) that can integrate known protein-protein correlation data to build upstream and downstream relationships between proteins [24]. The Common DEGs were inserted into STRING software to build and visualize the protein-protein interaction (PPI) network. Also, cytoHubba in Cytoscape software (Cytoscape_v3.6.1) was utilized to screen hub genes. The top 22 genes with a connection degree of > 5 were selected as hub genes.

### Drawing the ROC curve of Hub Genes

Using the package pROC, Receiver Operating Characteristic (ROC) curve analysis was performed on 22 hub genes. AUC > 90% was set as the cutoff value to determine the diagnostic significance of Hub Genes.

### Survival and statistical analysis

For survival analysis, gene expression values were divided into low and high expression groups by using R language. The hazard ratio (HR) was determined via a Cox regression model and survival curves were plotted from Kaplan-Meier estimations. *P* < 0.05 was considered to indicate a statistically significant difference.

### Hub Gene expression

The package ggpubr was used to draw a boxplot to observe the distribution of Hub Genes in liver cancer tissue and normal liver tissue.

### Gene set enrichment analysis

Gene set enrichment analysis (GSEA) is a computational method that assesses whether a prior defined set of genes shows statistical significance and concordant differences between two biological states [25]. To investigate the role of ESR1, SPP1 and FOBS gene in liver cancer, the package clusterProfiler was used to conduct single-gene GSEA analysis. *P*-value < 0.05 and p.adjust < 0.05 were regarded as the cut-off criteria.

## Results

### Identification of DEGs

**Fig. 1:**
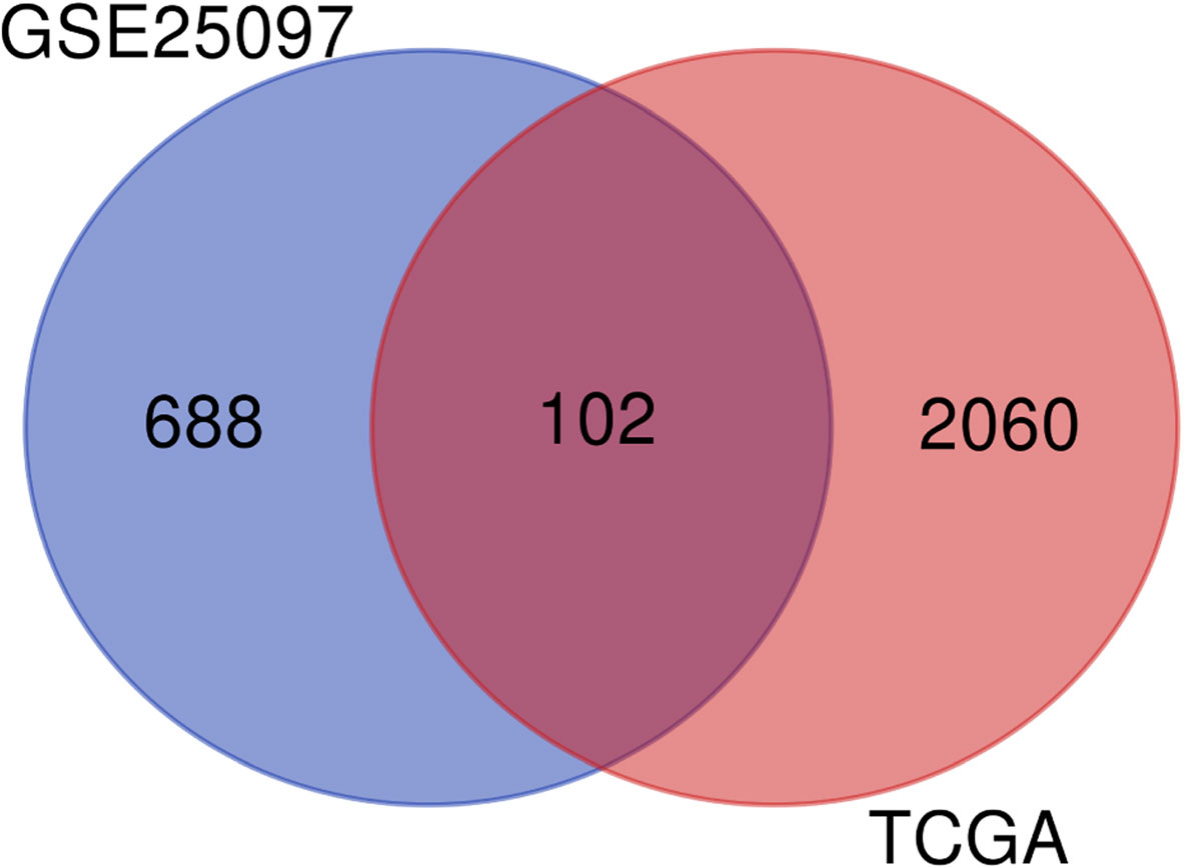
Venn diagram of DEGs of GSE25097 and TCGA LIHC datasets

**Fig. 2:**
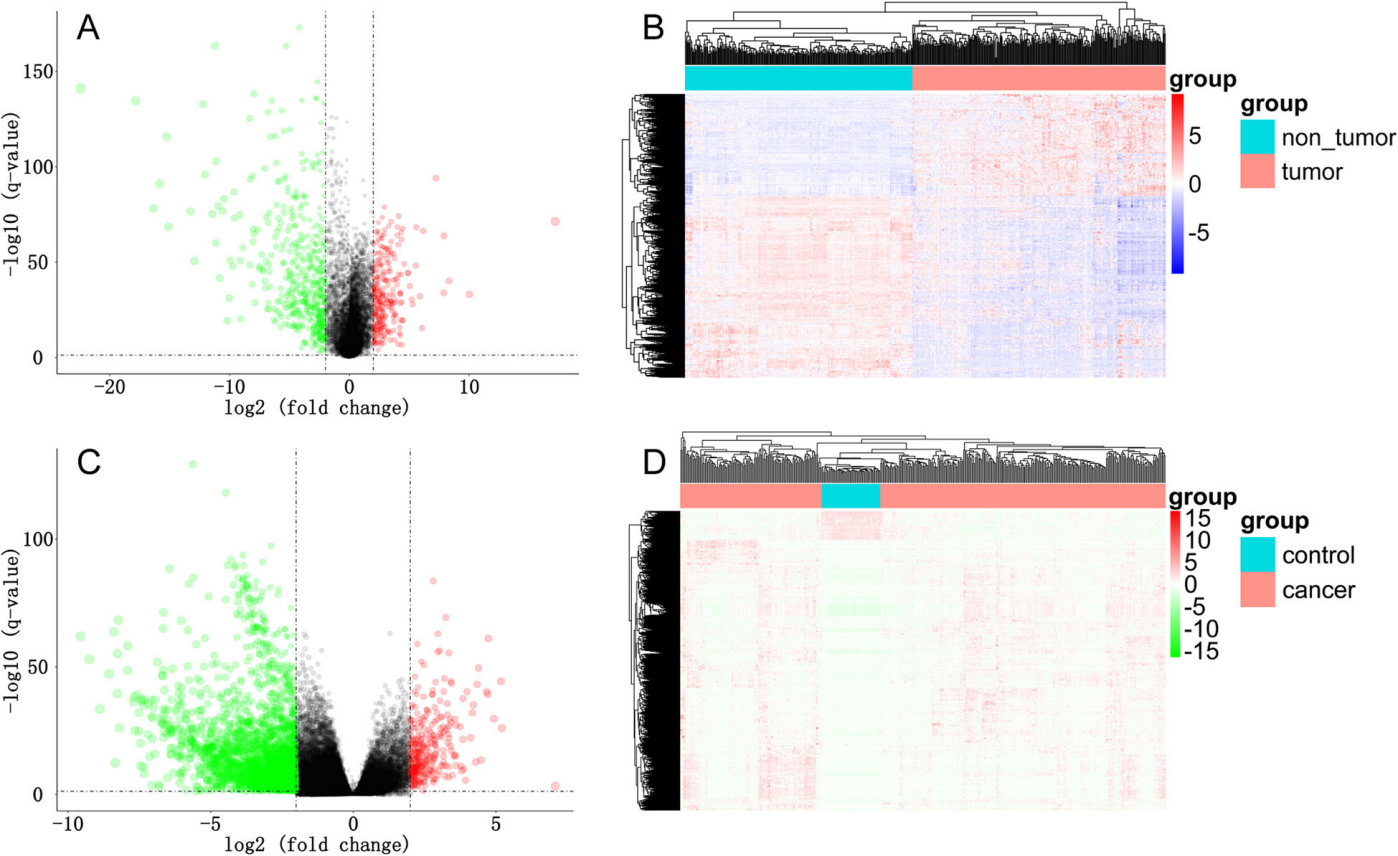
Identification of DEGs of GSE25097 and TCGA LIHC datasets, adj.*p*-value < 0.05 and |logFC| > 2 were used as the cut off criteria. LIHC: liver cancer; TCGA: The Cancer Genome Atlas. **A**. Volcano map of DEGs obtained from the GSE25097 dataset **B**. Heap map of DEGs obtained from the GSE25097 dataset **C**. Volcano map of DEGs obtained from the TCGA LIHC dataset **D**. Heat map of DEGs obtained from the TCGA LIHC dataset

The GSE25097 dataset was processed with R, DEGs with adj.*p* value < 0.05 and |logFC| > 2. This summed 790 genes, which were screened for further investigation (Fig. 1, Supplement Table 1). The TCGA LIHC dataset was analyzed with R × 64 3.6.1, using the package DEGseq2, Adj. *p* value < 0.05 and |logFC| > 2 were regarded as the cut-off criteria. This identified 2162 genes met the standards (Fig. 1, Supplement Table 2). To confirm the reliability of DEGs in liver cancer, Common DEGs of the two datasets were obtained which included 102 genes (Fig. 1, Table 1). The volcano map (Fig. 2A, Fig. 2C) and heat map (Fig. 2B, Fig. 2D) were drawn based on the differential genes obtained from data sets GSE25097 and TCGA LIHC, respectively..

**Table 1:** 102 Common DEGs in TCGA and GSE25097

Number	Gene
1	DCN
2	MARCO
3	ACSL4
4	CD5L
5	IGF2BP2
6	UBE2T
7	AFP
8	C1QTNF3
9	CETP
10	AURKA
11	ESR1
12	GSTZ1
13	PAGE4
14	CLEC4M
15	HAMP
16	CXCL12
17	MDK
18	GYS2
19	GMNN
20	GHR
21	THBS4
22	LIFR
23	C9
24	VIPR1
25	REG1A
26	RND3
27	HAO2
28	SPP1
29	NR4A3
30	EGR1
31	SOCS2
32	PCK1
33	MT1G
34	FOSB
35	PZP
36	ZFP36
37	GNAZ
38	DUSP9
39	TOP2A
40	LYVE1
41	MRO
42	STAB2
43	IGF2BP3
44	IL1RN
45	LRRC1
46	NUSAP1
47	CYP2C8
48	OIT3
49	COL2A1
50	PHLDA1
51	CYP1A2
52	FCN3
53	ECM1
54	PLAC8
55	CRHBP
56	CXCL14
57	GPC3
58	LCN2
59	CYP17A1
60	CNDP1
61	SPARCL1
62	NAT2
63	RCAN1
64	FCN2
65	PDZK1IP1
66	DNASE1L3
67	S100P
68	NPY1R
69	SPINK1
70	PTTG1
71	FBP1
72	CLEC1B
73	NNMT
74	GLYATL1
75	MFSD2A
76	ROBO1
77	FOS
78	HSD17B13
79	RRM2
80	REG3A
81	MUC13
82	SLC22A1
83	UBE2C
84	APOF
85	NQO1
86	SOCS3
87	SLC22A10
88	DLK1
89	CYP4A11
90	MT1X
91	FAM180A
92	IL1RAP
93	BCO2
94	AKR1B10
95	ADH4
96	SULT1C2
97	MT1F
98	COL15A1
99	MT1M
100	INMT
101	GBA3
102	CCL23

### GO and Reactome pathway analysis of the DEGs

**Fig. 3:**
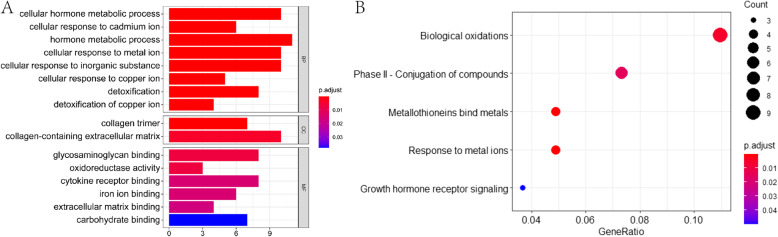
Enrichment analysis diagram of differentially expressed genes DEGs. **A**.GO analysis. **B**. Reactome analysis

GO analysis and Reactome Pathway analysis were used to conduct enrichment analysis of the 102 Common DEGs. GO analysis included biological process (BP), cellular component (CC) and molecular function (MF) analysis (Fig. 3a). BP analysis showed that liver cancer caused changes in hormone metabolism (Cellular hormone metabolic process, Hormone metabolic process), cell reaction to copper, cadmium ions and inorganic substances and detoxification function (Cellular response to cadmium ion, Cellular response to metal ion, Cellular response to inorganic substance, Cellular response to copper ion, Detoxification of copper ion and Detoxification). CC analysis showed that the Collagen trimer and Collagen-containing extracellular matrix of liver cancer cells were changed. Moreover, the MF analysis showed that patients with liver cancer had an abnormal expression of oxidoreductase activity and molecular binding function (Glycosaminoglycan binding, Cytokine receptor binding, iron ion binding, extracellular matrix binding and carbohydrate binding). The results showed that the changes of collagen were observed at the cellular level. The changes of hormone metabolism, reaction to metal ions and detoxification were observed at the biological function leved and the changes of molecular binding and oxidoreductase activity were observed at the molecular level.

Through Reactome enrichment analysis (Fig. 3B), it was seen that liver cancer caused changes in biological oxidation reactions and conjugation ability to metal ions (phase II-conjugation of compounds, metallothioneins bind metals and response to metal ions) and also affected growth hormone receptor signaling.

Comparing the results of the two enrichment analyses, it was found that the information obtained by the two was consistent. The two analyses were enriched with changes in hormone metabolism, biological oxidation, cell reaction to metal ions and other aspects in patients with liver cancer.

### PPI network analysis and screening for Hub Genes

**Fig. 4:**
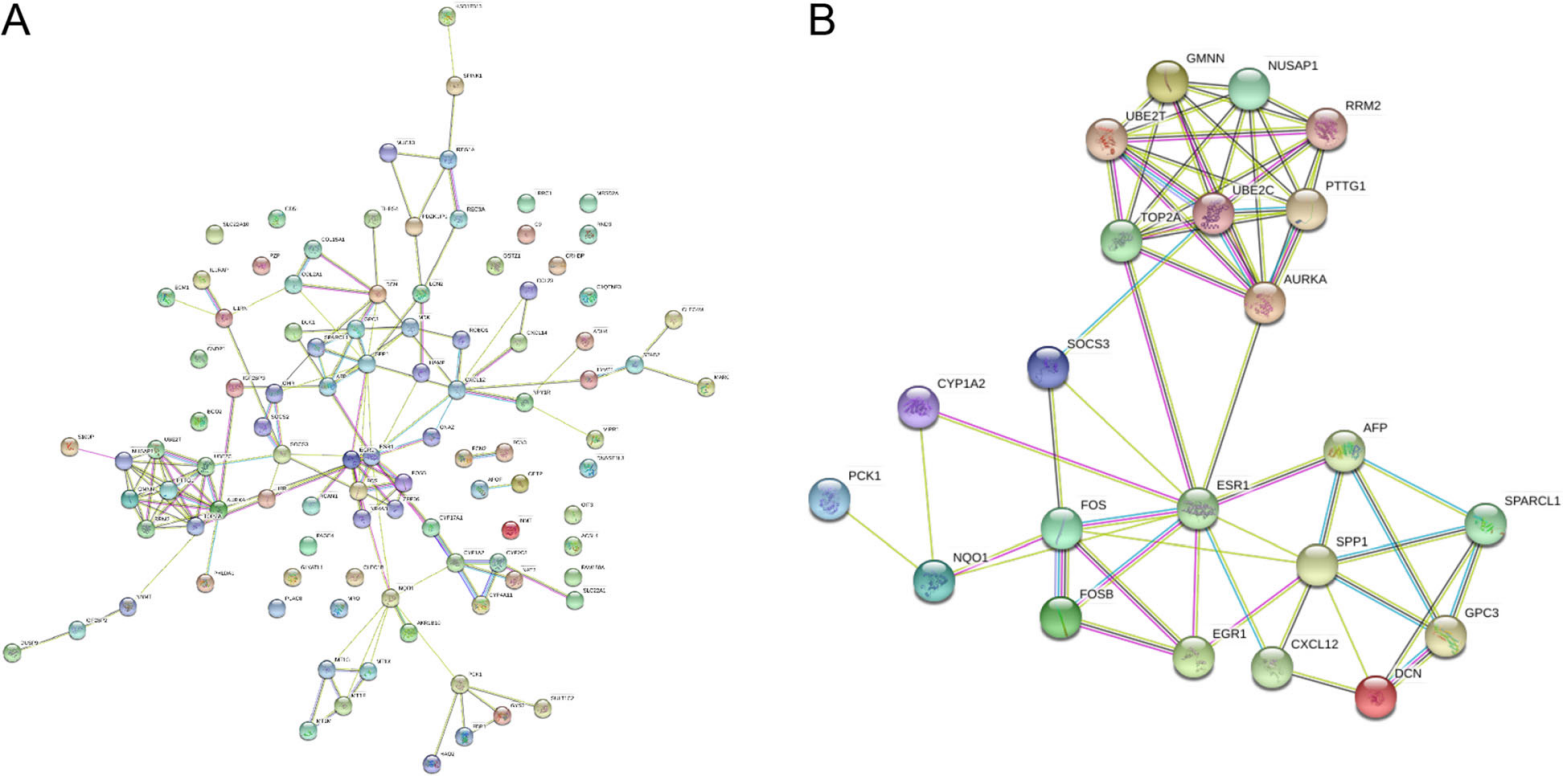
PPI network diagram drawn by String. **a**. PPI network map of 102 DEGs. **b**. PPI network map of 22 Hub Genes

102 DEGs were used as input into STRING to build a PPI network (Fig. 4A). The PPI network diagram was exported to Cytoscape (3.2.1). CytoHubba app plug-in was used to calculate the Degree Value and other parameter values (Supplement Table 3). Genes whose Degree Values are > = 5 are taken as Hub Genes and a total of 22 Hub Genes were obtained (Table 2). See Fig. 4B for the relationship between 22 Hub Genes.

**Table 2:** Top 22 Hub Genes with degree > = 5

Number	Name	Betweenness	BottleNeck	Closeness	Clustering Coefficient	Degree	DMNC	EcCentricity	EPC	MCC	MNC	Radiality	Stress
1	ESR1	2780.97619	58	38.78333	0.16667	13	0.2036	0.15823	29.27	28	11	8.416	5698
2	SPP1	1519.02063	30	35.65	0.22727	12	0.2545	0.18987	28.52	35	11	8.1723	3920
3	AURKA	967.42857	11	32.02619	0.4	11	0.6415	0.13562	24.93	5045	8	7.8002	1918
4	CXCL12	1415.00476	15	32.73333	0.11111	9	0.2842	0.15823	24.1	11	4	7.9926	2458
5	FOS	617.74127	17	33.3	0.30556	9	0.3207	0.15823	27.17	29	8	8.0311	1918
6	NQO1	1353	11	31.55952	0.17857	8	0.309	0.13562	22.39	12	3	7.8515	2882
7	NUSAP1	146	2	25.78452	0.75	8	0.7683	0.11867	23.93	5041	7	7.0561	338
8	TOP2A	332.07302	3	30.27619	0.78571	8	0.6415	0.13562	24.54	5042	8	7.7361	938
9	UBE2C	146.37619	1	27.25119	0.75	8	0.7683	0.11867	24.36	5041	7	7.2229	264
10	AFP	281.74603	4	30.05	0.33333	7	0.3328	0.15823	26.15	17	6	7.7874	770
11	DCN	303.6	3	28.55	0.28571	7	0.2853	0.15823	24.75	13	6	7.5308	782
12	EGR1	228.02381	3	30.21667	0.42857	7	0.4279	0.15823	25.63	25	6	7.8002	602
13	GMNN	0	1	25.28452	1	7	0.7683	0.11867	24.18	5040	7	7.0433	0
14	PTTG1	0	1	25.28452	1	7	0.7683	0.11867	23.95	5040	7	7.0433	0
15	RRM2	0	1	25.28452	1	7	0.7683	0.11867	23.64	5040	7	7.0433	0
16	SOCS3	631.69841	6	31.05952	0.09524	7	0.3078	0.13562	25.03	7	2	7.8259	1378
17	UBE2T	0	1	25.28452	1	7	0.7683	0.11867	24.05	5040	7	7.0433	0
18	CYP1A2	577	5	29.39286	0.2	6	0.309	0.13562	19.57	7	3	7.7361	822
19	GPC3	44.47619	2	26.7	0.53333	6	0.3804	0.15823	24.62	20	6	7.364	138
20	FOSB	35.46667	1	27.55952	0.7	5	0.4538	0.13562	24.25	18	5	7.5821	166
21	PCK1	570	5	24.21786	0.1	5	0.3078	0.11867	10.7	5	2	7.0176	1074
22	SPARCL1	18.43333	1	26.53333	0.6	5	0.389	0.15823	24.88	14	5	7.3897	62

### Expression of Hub Genes in patients with liver cancer

**Fig. 5:**
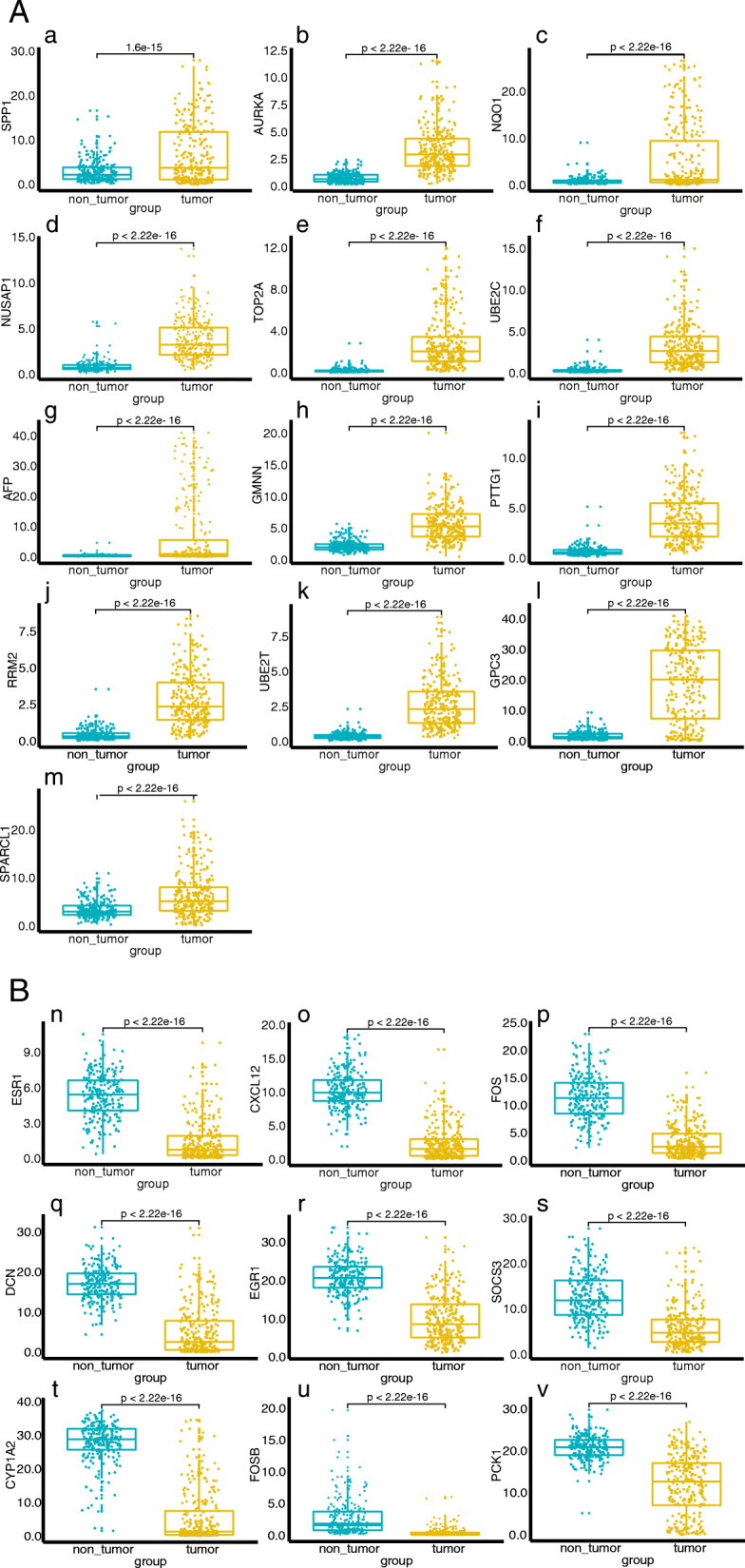
Expression levels of 22 Hub Gene. **A**. genes that is highly expressed in liver cancer. (**a**) SPP1, (**b**) AURKA, (**c**) NQO1, (**d**) NUSAP1, (**e**) TOP2A, (**f**) UBE2C, (**g**) AFP, (**h**) GMNN, (**i**) PTTG1, (**j**) RRM2, (**k**) UBE2T, (**l**) GPC3, (**m**) SPARCL1 in Normal Liver versus Liver Cancer tissues. **B**. genes that is lowly expressed in liver cancer. (**n**) ESR1, (**o**) CXCL12, (**p**) FOS, (**q**) DCN, (**r**) EGR1, (**s**) SOCS3, (**t**) CYP1A2, (**u**) FOSB, (**v**) PCK1 in Normal Liver versus Liver Cancer tissues

The expression of 22 Hub Genes in liver cancer and normal liver tissues was analyzed and it was found that SPP1, AURKA, NQO1, NUSAP1, TOP2A, UBE2C, AFP, GMNN, PTTG1, RRM2, UBE2T, GPC3, SPARCL1 etc. (Fig. 5A), a total of 13 genes, were highly expressed in liver cancer tissues. However, ESR1, CXCL12, FOS, DCN, EGR1, SOCS3, CYP1A2, FOSB, PCK1 etc., a total of 9 genes, were under-expressed in liver cancer tissues (Fig. 5B).

### ROC curve analysis of Hub Genes

**Fig. 6:**
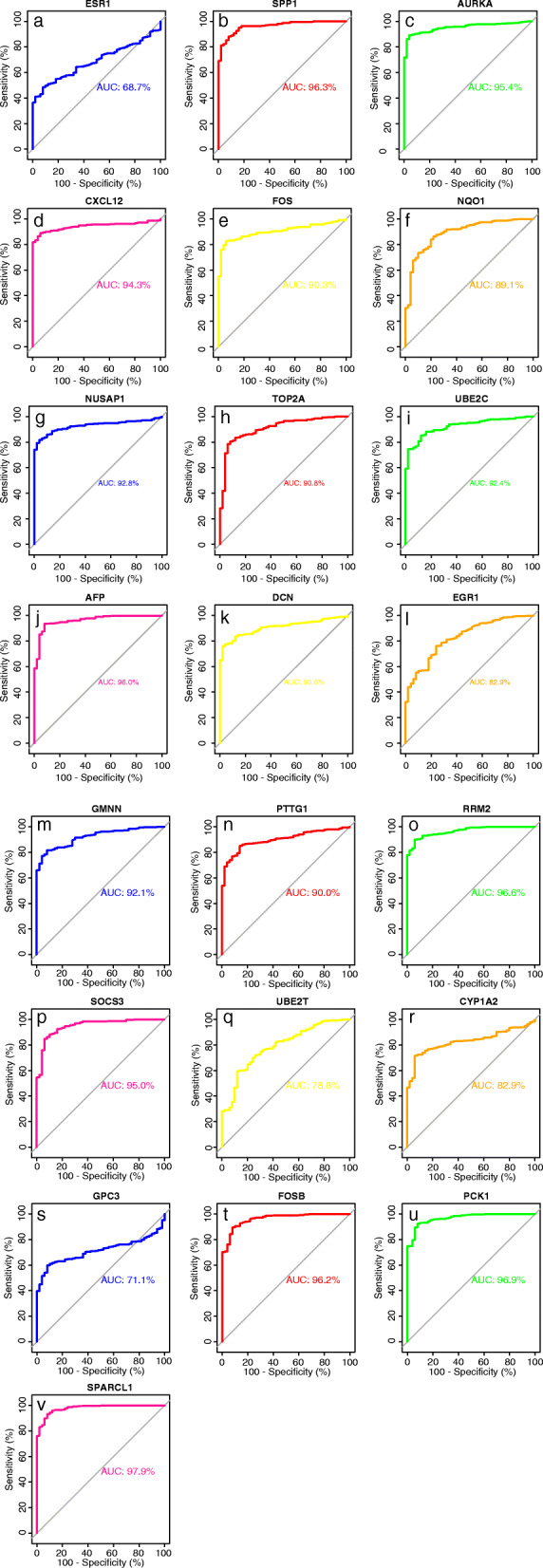
ROC curve of Hub Gene. **a** ESR1, (**b**)SPP1, (**c**) AURKA, (**d**)CXCL12, (**e**) FOS, (**f**) NQO1, (**g**) NUSAP1, (**h**)TOP2A, (**i**)UBE2C, (**j**) AFP, (**k**) DCN, (**l**) EGR1, (**m**) GMNN, (**n**)PTTG1, (**o**)RRM2, (**p**)SOCS3, (**q**)UBE2T, (**r**)CYP1A2, (**s**)GPC3, (**t**) FOSB, (**u**)PCK1, (**v**)SPARCL1

ROC curve analysis was performed on 22 Hub Genes using the package pROC. AUC > 90% was taken as the cutoff value, and it was found that 16 of the 22 Hub Genes with AUC > 90% included SPP1, AURKA, CXCL12, FOS, NUSAP1, TOP2A, UBE2C, AFP, DCN, GMNN, PTTG1, RRM2, SOCS3, FOSB, PCK1 and SPARCL1 respectively. The expression levels of these genes have high accuracy in distinguishing normal tissue from liver cancer tissue, and could be a potential “tumor biomarker”. At the same time, it can be used as a biomarker for the diagnosis of liver cancer, which has important significance for the accurate diagnosis of liver cancer (Fig. 6).

### The survival curve of Hub Genes

**Fig. 7:**
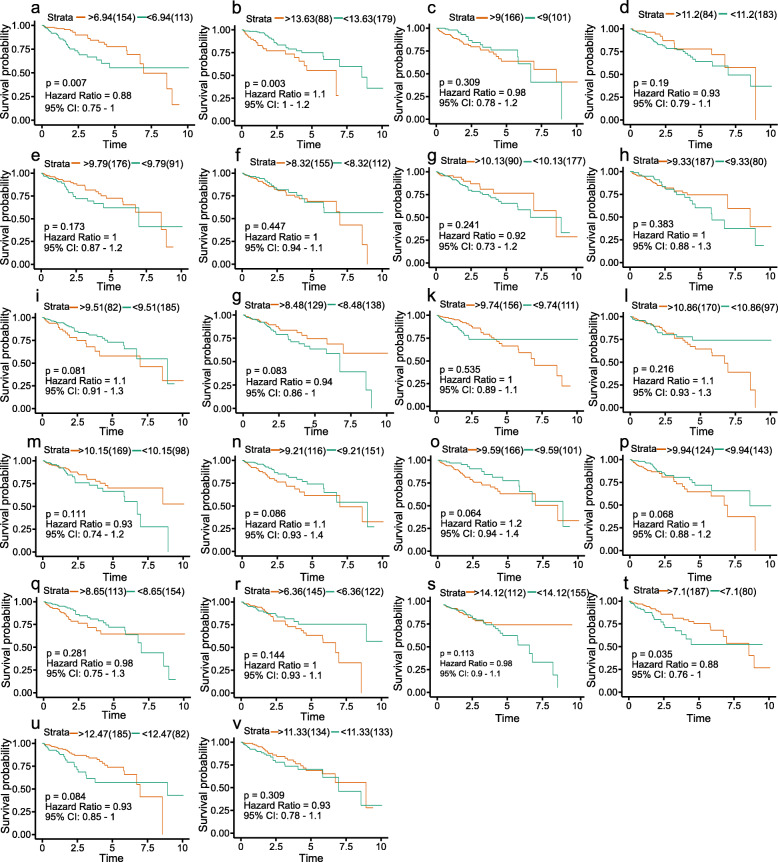
Survival analysis of 22 Hub Genes: (**a**) ESR1, (**b**) SPP1, (**c**) AURKA (**d**) CXCL12, (**e**) FOS, (f) NQO1, (**g**) NUSAP1, (**h**) TOP2A, (**i**) UBE2C, (**j**) AFP, (**k**) DCN, (**l**) EGR1, (**m**) GMNN, (**n**) PTTG1, (**o**) RRM2, (**p**) SOCS3, (**q**) UBE2T, (**r**) CYP1A2, (**s**) GPC3, (**t**) FOSB, (**u**) PCK1, (**v**) SPARCL1; *p* < 0.05 was considered as statistically significant

Survival curves were plotted from Kaplan-Meier estimations (Fig. 7). The Cox regression model was used to calculate the Hazard Ratio (HR) of the hub genes for liver cancer patients. The results showed that among these Hub Genes, the expression levels of ESR1, SPP1 and FOSB genes was closely related to the survival time of liver cancer patients, with statistically significant differences (*p* < 0.05). HR values were 0.88, 1.1 and 0.88, respectively, This can be translated as ESR1 and FOSB representing low-risk factors, while SPP1 was a high-risk factor.

### GSEA revealed the biological function that affects the survival time of liver cancer

**Fig. 8:**
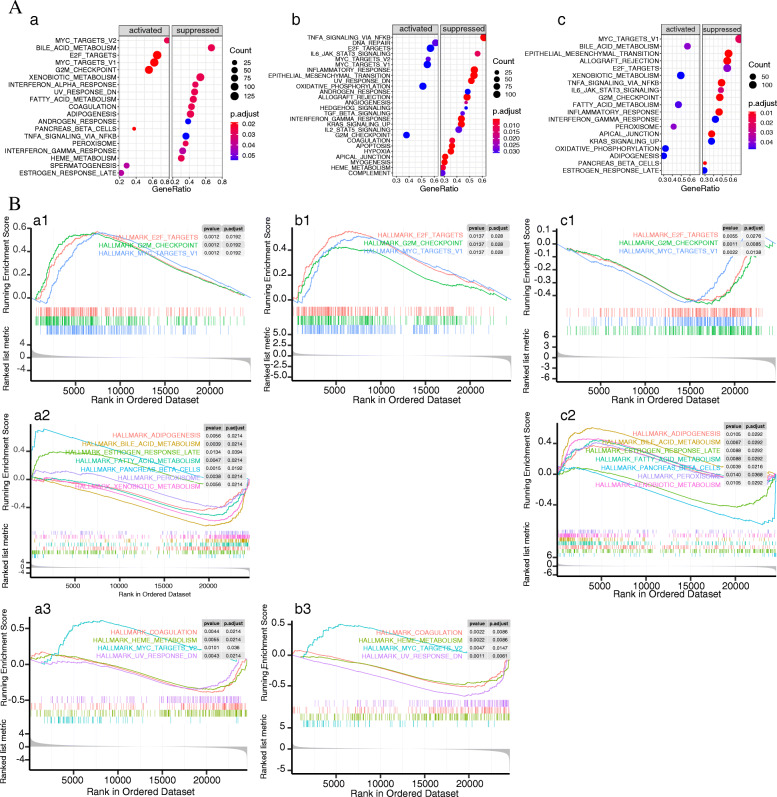
Identification of the enriched gene sets with GSEA analysis focused on a single gene as a phenotype. **A**.dot plot. **B**.curve graph. **a1**,**b1** and **c1** are the common pathways obtained by enrichment of ESR1, FOSB and SSP1 genes, respectively; **a2** and **c2** are the common pathways obtained by enrichment of ESR1 and SSP1 genes, respectively; **a3** and **b3** are the common pathways obtained by enrichment of ESR1 and FOSB genes, respectively

Single-gene GSEA was used to investigate biological pathways and biological functions related to survival time (Fig. 8). Figure 8A shows all the related pathways of ESR1, FOSB and SSP1 genes,respectively. Figure 8B shows the commonly related pathways of ESR1, FOSB and SSP1 genes. Figure 8B a1, b1 and c1 are the three common pathways of ESR1, FOBS and SPP1 genes. Figure 8B a2 and c2 are the seven common pathways of ESR1 and SPP1 genes, Fig. 8B a3 and b3 are the four common pathways of ESR1 and FOSB genes.

The three common pathways enriched by ESR1, FOBS and SPP1 genes are HALLMARK MYC TARGETS V1, HALLMARK G2M CHECKPOINT and HALLMARK E2F TARGETS pathways (Fig. 8Ba1, b1, c1). According to the information in Fig. 8B, it can be seen that the high expression of ESR1 and FOBS can activate these three pathways, while the high expression of SPP1 can inhibit these three pathways. However, in liver cancer tissue, ESR1 and FOBS genes were low in expression, while SPP1 genes were high in expression (see Fig. 5). Therefore, changes in the expression levels of ESR1, FOBS and SPP1 genes in liver cancer inhibited all three pathways.

Seven common pathways were obtained by enrichment analysis of ESR1 and SPP1 genes. They are HALLMARK PANCREAS BETA CELLS, HALLMARK ESTROGEN RESPONSE LATE, HALLMARK ADIPOGENESIS, HALLMARK FATTY ACID METABOLISM, HALLMARK BILE ACID METABOLISM, HALLMARK XENOBIOTIC METABOLISM and HALLMARK PEROXISOME pathways. The high expression of the ESR1 gene can activate the HALLMARK PANCREAS BETA CELLS and HALLMARK ESTROGEN RESPONSE LATE pathways, and five pathways, namely, HALLMARK ADIPOGENESIS, HALLMARK FATTY ACID METABOLISM, HALLMARK BILE ACID METABOLISM, HALLMARK XENOBIOTIC METABOLISM, and HALLMARK PEROXISOME were inhibited, while the SPP1 gene was opposite to the ESR1 gene (Fig. 8Ba2, c2). In liver cancer, the ESR1 gene is a low expression gene, while the SPP1 gene is a high expression gene (see Fig. 5). Therefore, changes in ESR1 and SPP1 gene expression in liver cancer activated the HALLMARK ADIPOGENESIS, HALLMARK FATTY ACID METABOLISM, HALLMARK BILE ACID METABOLISM, HALLMARK XENOBIOTIC METABOLISM, and HALLMARK PEROXISOME pathways. However both the HALLMARK PANCREAS BETA CELLS and HALLMARK ESTROGEN RESPONSE LATE pathways were suppressed.

The four common pathways enriched by ESR1 and FOSB genes are HALLMARK MYC TARGETS V2, HALLMARK HEME METABOLISM, HALLMARK COAGULATION and HALLMARK UV RESPONSE DN pathways. High expression of ESR1 and FOSB can activate the HALLMARK MYC TARGETS V2 pathway and inhibit three pathways, namely HALLMARK HEME METABOLISM, HALLMARK COAGULATION and HALLMARK UV RESPONSE_DN (Fig. 8B a3, b3). However, in liver cancer, both ESR1 and FOBS genes were low expressed (see Fig. 5). Therefore, the changes in the expression levels of ESR1 and FOBS genes in liver cancer inhibited HALLMARK MYC TARGETS V2 pathway, while HALLMARK HEME METABOLISM, HALLMARK COAGULATION and HALLMARK UV RESPONSE_DN pathways were activated.

## Discussion

Most patients with liver cancer do not seek medical treatment until they have symptoms in the late stage of liver cancer, therefore the early diagnosis of liver cancer is of great significance for treatment. At present, alpha-fetoprotein (AFP) is a diagnostic biomarker used in the clinical diagnosis of liver cancer. AFP was discovered 50 years ago as a diagnostic biomarker of liver cancer and currently, there are problems associated with the inaccuracy of diagnosis. According to investigations, 32 to 59% of liver cancer patients have normal AFP levels [22]. Therefore, it is necessary to find new and more accurate biomarkers for liver cancer diagnosis. Also, the prognosis of cancer patients is of great significance to the quality of life and treatment of patients. Therefore, the search for prognostic biomarkers is also of great significance for tumor patients. In order to achieve this goal, this scientific work uses data mining analysis to find diagnostic biomarkers and prognostic biomarkers associated with liver cancer.

First, liver cancer data sets from the TCGA database were obtained which included 50 normal liver tissue samples and 371 liver cancer samples. The GSE25097 dataset was obtained from the GEO database consisted of 243 non-tumor tissue samples and 268 liver cancer samples. After DEGs analysis, 102 Common DEGs were obtained from TCGA and GSE25097 data sets. GO analysis was then conducted and Reactome Pathway analysis was used to conduct enrichment analysis on 102 Common DEGs, The results showed that liver cancer showed changes in collagen at the cellular level, changes in hormone metabolism and reaction to metal ions at the biological function and abnormalities in molecular binding and oxidoreductase activity at the molecular level (Fig. 3).

A PPI network was constructed for 102 Common DEGs to find the correlation between genes and 22 Hub Genes were screened from 102 Common DEGs based on Degree value (Table 2). ROC curve is a curve reflecting the relationship between sensitivity and specificity, which is of great significance for the accurate diagnosis of diseases [26]. A ROC curve was used to analyze 22 Hub Genes with AUC greater than 90% as the threshold and this resulted in 16 Hub Genes. They were SPP1, AURKA, CXCL12, FOS, NUSAP1, TOP2A, UBE2C, AFP, DCN, GMNN, PTTG1, RRM2, SOCS3, FOSB, PCK1 and SPARCL1. The expression levels of the 16 Hub Genes in liver cancer can accurately distinguish normal liver tissue from liver cancer, therefore the 16 genes can be used as diagnostic biomarkers of liver cancer for the early diagnosis of liver cancer (along with AFP which is currently used in clinical practice). At the same time, the effect of the 22 Hub Ggenes on the survival time of liver cancer patients was observed and the risk coefficient was calculated. It was found that the expression levels of ESR1, SPP1 and FOSB genes in the 22 hub genes had a significant impact on the survival time of liver cancer patients(*p* < 0.05), with HR values of 0.88, 1.1 and 0.88, respectively, indicating that ESR1 and FOSB are low-risk genes while SPP1 is high-risk gene. However, the AUC value of ESR1 is 68.7%(Fig. 6a), which showed that the accurate diagnosis rate of ESR1 gene is low and not suitable for use as a diagnostic biomarker. As a result, only the FOSB and SPP1 genes are suitable for use as prognostic biomarkers of liver cancer, where the FOSB is a low-risk gene while the SPP1 is a high-risk gene. In other words, the survival rate of liver cancer patients with high expression of FOSB is higher than that of patients with low expression. In comparison, the survival rate of patients with high expression of SPP1 is lower than that of patients with low expression. This conclusion has been verified through literature. Tang C. et al. found that an overexpression of FOSB protein inhibited tumor cell proliferation, clone formation and cell migration [27], while the silencing of FOSB protein expression promoted tumor cell proliferation, clone formation and cell migration [28]. Li H.’s study also confirmed that the overexpression of FOSB protein can promote the proliferation of cancer cells. These studies confirmed that FOSB is a low-risk gene. Similarly, regarding SPP1, Lu C et al. found that the silencing of OPN protein (encode by SPP1 gene) in liver cancer reduced the number of cell clones and proliferation rate, and in vivo pharmacodynamics observed that the tumor volume of tumor-bearing mice decreased [29]. It was confirmed that the SPP1 is a high-risk gene.

Finally, single-gene GSEA analysis was performed on the three prognostic genes, ESR1, SPP1 and FOSB, that affect the survival time of liver cancer patients (Fig. 8) in order to explore the mechanism affecting the prognosis of liver cancer patients. Through analysis, it was found that there were three pathways closely related to ESR1, FOBS and SPP1 genes (Fig. 8B a1, b1, c1), seven pathways closely related to ESR1 and SPP1 genes (Fig. 8B a2, c2), and four pathways closely related to ESR1 and FOSB genes (Fig. 8B a3, b3).

The three common pathways related to ESR1, FOBS, and SPP1 genes are HALLMARK MYC TARGETS V1, HALLMARK G2/M CHECKPOINT and HALLMARK E2F TARGETS. Among them, high expression of ESR1 and FOBS genes can activate these three pathways, while high expression of SPP1 gene inhibits these three pathways (Fig. 8a1, b1, c1). At the same time, since ESR1 and FOBS genes are low-risk factors, high expression of ESR1 and FOBS genes can activate these three pathways. SPP1 gene is a high-risk factor, high expression of SPP1 can inhibit these three pathways (Fig. 8 a, b, t). Hence, activation of these three pathways is conducive to improving the survival time of liver cancer patients. MYC TARGETS V1 pathway is a new anticancer target [30–32] which is closely related to cell proliferation, differentiation and cell cycle. In contrast, the G2/M CHECKPOINT pathway [33] and HALLMARK E2F TARGETS pathway are all closely related to the cell cycle [34]. In summation, patients with liver cancer whose cell cycle pathway is activated have a better prognosis.

The seven common pathways related to ESR1 and SPP1 genes are HALLMARK PANCREAS BETA CELLS, HALLMARK ESTROGEN RESPONSE LATE, HALLMARK ADIPOGENESIS, HALLMARK FATTY ACID METABOLISM, HALLMARK BILE ACID METABOLISM, HALLMARK XENOBIOTIC METABOLISM and HALLMARK PEROXISOME. Among them, high ESR1 gene expression can activate the HALLMARK PANCREAS BETA CELLS and HALLMARK ESTROGEN RESPONSE LATE pathways, inhibit the five pathways of HALLMARK ADIPOGENESIS, HALLMARK FATTY ACID METABOLISM, HALLMARK BILE ACID METABOLISM, HALLMARK XENOBIOTIC METABOLISM and HALLMARK PEROXISOME. In contrast, SPP1 gene was opposite to ESR1 gene (Figure 8 a2, c2). Similarly, the ESR1 gene represents a low-risk-factor, SPP1 gene represent a high-risk factor and therefore liver cancer patients that show HALLMARK PANCREAS BETA CELLS and HALLMARK ESTROGEN RESPONSE LATE pathway activated and the HALLMARK ADIPOGENESIS, HALLMARK FATTY ACID METABOLISM, HALLMARK BILE ACID METABOLISM, HALLMARK XENOBIOTIC METABOLISM and HALLMARK PEROXISOME pathways inhibited have a better prognosis. By analyzing these pathways, it has been found that these seven pathways can be divided into four aspects in terms of function: 1. The prognosis of liver cancer patients with HALLMARK PANCREAS BETA CELLS pathway activated is better than that of liver cancer patients with this pathway inhibited. HALLMARK PANCREAS BETA CELLS pathway restrained and islet cell dysfunction are important cause of type 2 diabetes. This also means that patients with liver cancer complicated with type 2 diabetes have a poor prognosis. Patients with type 2 diabetes are also a high-risk population for developing liver cancer. This conclusion is consistent with the conclusion of an epidemiological investigation of liver cancer [17]. 2. The prognosis of liver cancer patients that HALLMARK ESTROGEN RESPONSE LATE pathway activated is better. Clinically, “Palmar Erythema” and “spider nevus” appear in the palms of some patients with cancer [35] and severe liver dysfunction [36]. These manifestations are caused by the decreased metabolism of estrogen in the liver, resulting in excessive estrogen [37] in the blood and stimulation of capillary arterial congestion and dilation. In other words, the presence of “Palmar Erythema” and “spider arachnoid” is a manifestation of the inhibition of estrogen pathway and the prognosis of liver cancer patients with “ Palmar Erythema “ and “ spider nevus “ is poor. Also, in clinical practice, some male liver cancer patients, due to the inhibition of estrogen metabolism, have an increase of estrogen level in their blood resulting in breast development. The prognosis of such liver cancer patients is not positive [38]. 3. The prognosis is better in patients with liver cancer whose fat metabolism-related pathways (HALLMARK ADIPOGENESIS, HALLMARK FATTY ACID METABOLISM, HALLMARK BILE ACID METABOLISM and HALLMARK PEROXISOME) are inhibited. Epidemiological investigation shows that obesity is one of the important factors causing liver cancer and for the prognosis of liver cancer patients, the prognosis of patients with fat metabolism-related pathways being inhibited is better. 4. Patients whose HALLMARK XENOBIOTIC METABOLISM is inhibited have a more positive prognosis.

Four common pathways related to ESR1 and FOSB genes are activation of HALLMARK MYC TARGETS V2 and inhibition of HALLMARK HEME METABOLISM, HALLMARK COAGULATION and HALLMARK UV RESPONSE DN pathways. Both ESR1 and FOSB genes were low-risk factors, therefore patients whose HALLMARK MYC TARGETS V2 pathway was activated, and the HALLMARK HEME METABOLISM, HALLMARK COAGULATION and HALLMARK UV RESPONSE DN pathways were suppressed had a better prognosis. HALLMARK E2F TARGETS V2 pathway is closely related to the cell cycle, that is to say, the prognosis of liver cancer patients with activated cell cycle pathway is better, which is consistent with the conclusion previously arrived at. Also, HALLMARK HEME METABOLISM pathway regulates HEME METABOLISM, and the main product of HEME METABOLISM is bile pigment, which includes many compounds such as bilirubin, biliverdin, bilinogen and choline. Under normal circumstances, bile pigment is mainly excreted with bile. Bilirubin is the main pigment in bile, which is orange-yellow in color. The metabolic disorder of bilirubin is closely related to clinical hepatobiliary diseases. If the HALLMARK HEME METABOLISM pathway is activated, the heme will be massively metabolized into bilirubin, resulting in an excessively high concentration in plasma and then will be diffused into tissue, resulting in jaundice (easily seen in sclera, skin, etc.). According to the conclusions of the data analysis in this scientific work, patients with inhibited HALLMARK METABOLISM pathway have a good prognosis. In contrast, those with an activated HALLMARK METABOLISM pathway have a poor prognosis. After having activated HALLMARK METABOLISM pathway, patients will show jaundice related symptoms and liver cancer patients with jaundice have a poor prognosis whilepatients with suppressed HALLMARK COAGULATION pathway have a good prognosis, The HALLMARK COAGULATION pathway mainly regulates the COAGULATION function. Abnormal COAGULATION function in liver cancer patients is a common clinical symptom, mainly related to the lack of COAGULATION factor, thrombocytopenia and increased vascular permeability. The results of the data analysis in this work show that the prognosis of patients with inhibited blood clotting function is better than that of patients with this function activated.

Through a very detailed and painstkeing analysis, it was found that the prognosis of liver cancer patients is mainly related to the following functions: 1. It is closely related to the regulation of the cell cycle and patients with activated cell cycle have a good prognosis. 2. Liver cancer patients with activated HALLMARK PANCREAS BETA CELLS pathway have a good prognosis, while liver cancer patients with type 2 diabetes have a poor prognosis. 3. Patients with activated hepatocellular estrogen pathway have a good prognosis and those with “liver palm”, “spider nevus” and abnormal breast development have a poor prognosis. 4. Liver cancer patients whose fat metabolism-related pathways are inhibited have a good prognosis. 5. Liver cancer patients whose HALLMARK XENOBIOTIC METABOLISM pathway is inhibited have a good prognosis. 6. The prognosis of liver cancer patients is good if HALLMARK HEME METABOLISAM pathway is inhibited, and poor if the patient has “jaundice”. 7. Liver cancer patients whose HALLMARK COAGULATION pathway is inhibited have a good prognosis.

## Conclusion

Ten genes have been identified which show high expression in the event of liver cancer. These include SPP1, AURKA, NUSAP1, TOP2A, UBE2C, AFP, GMNN, PTTG1, RRM2 and SPARCL1. Six genes show low expression and include CXCL12, FOS, DCN, SOCS3, FOSB and PCK1. These can be used as markers for liver cancer diagnosis, among which FOBS and SPP1 genes can also be used as prognostic markers of liver cancer. Activation of the cell cycle-related pathway, PANCREAS BETA CELLS pathway and the estrogen signaling pathway in LIVER CANCER patients, while inhibition of the HALLMARK HEME METABOLISM pathway, HALLMARK COAGULATION pathway, and the fat metabolism pathway may promote prognosis in LIVER CANCER patients.

## Supplementary Information

**Additional file 1: Supplement Table 1.** The differentially expressed genes of GSE25097

**Additional file 2: Supplement Table 2.** TCGA the differentially expressed genes

**Additional file 3: Supplement Table 3.** Parameter values of the common differentially expressed genes

## Data Availability

The datasets generated and/or analyzed during the current study are available in the [https://www.ncbi.nlm.nih.gov/geo/database] and https://portal.gdc.cancer.gov/].

## Abbreviations

AFP: Alpha-fetoprotein
BP: Biological process
CC: Cellular component
Common DEGs: The common differentially expressed genes
DEGs: The differentially expressed genes
GEO: Gene expression omnibus
GSEA: Gene set enrichment analysis
HBV: Hepatitis B virus
HCV: Hepatitis C virus
HR: The hazard ratio
MF: Molecular function
ROC: Receiver operating characteristic curve
STRING: Search tool for the retrieval of interacting genes/proteins
TCGA: The cancer genome atlas
